# Acute stress induces severe neural inflammation and overactivation of glucocorticoid signaling in interleukin-18-deficient mice

**DOI:** 10.1038/s41398-022-02175-7

**Published:** 2022-09-23

**Authors:** Kyosuke Yamanishi, Nobutaka Doe, Keiichiro Mukai, Takuya Hashimoto, Naomi Gamachi, Masaki Hata, Yuko Watanabe, Chiaki Yamanishi, Hideshi Yagi, Haruki Okamura, Hisato Matsunaga

**Affiliations:** 1 Department of Neuropsychiatry, School of Medicine, Hyogo Medical University, Nishinomiya, Hyogo 6638501 Japan; 2 Department of Psychoimmunology, School of Medicine, Hyogo Medical University, Nishinomiya, Hyogo 6638501 Japan; 3 Laboratory of Neurogenesis and CNS Repair, Institute for Advanced Medical Sciences, Hyogo Medical University, Nishinomiya, Hyogo 6638501 Japan; 4 Department of Occupational Therapy, School of Rehabilitation, Hyogo Medical University, Nishinomiya, Hyogo 6638501 Japan; 5 Hirakata General Hospital for Developmental Disorders, Hirakata, Osaka, 5730122 Japan; 6 Department of Anatomy and Cell Biology, School of Medicine, Hyogo Medical University, Nishinomiya, Hyogo 6638501 Japan

**Keywords:** Molecular neuroscience, Depression

## Abstract

Interleukin-18 (IL18) is an inflammatory cytokine that is related to psychiatric disorders such as depression and cognitive impairment. We previously found that IL18 deficiency may cause hippocampal impairment, resulting in depression-like behavioral changes. However, the potential role of IL18 in stressful conditions remains uncertain. In the present study, we examined the effect of IL18 on neural inflammation and stress tolerance during acute stress. Littermate *Il18*^+/+^ and *Il18*^−/−^ mice were exposed to a single restraint stress for 6 h, and all assessments were performed 18 h after the mice were released from the restraint. In *Il18*^−/−^ mice exposed to acute stress, the immobility times in both the forced swim test and tail suspension test were decreased, although no difference was observed in *Il18*^*+/+*^ mice. *Il1β*, *Il6*, and *Tnfα* expression levels in the hippocampus of stressed *Il18*^−/−^ mice were significantly higher than those in the other groups. Moreover, the numbers of astrocytes and microglia, including those in the active form, were also increased compared with those in other groups. Regarding the molecular mechanism, the *HSF5* and *TTR* genes were specifically expressed in stressed *Il18*^−/−^ mice. As a potential treatment, intracerebral administration of IL18 to *Il18*^−/−^ mice resulted in partial recovery of changes in behavioral assessments. Our results revealed that IL18-deficient mice were more sensitive and had a longer response to acute stress than that in normal mice. In addition, neural inflammation and augmentation of glucocorticoid signals caused by stress were more intense and remained longer in *Il18*^−/−^ mice, resulting in behavioral changes. In conclusion, IL18 might be an indispensable factor that modulates the stress response and maintains balance between neural inflammation and glucocorticoid signaling.

## Introduction

In humans, stressful conditions are essentially universal, often cause severe problems, and may ultimately lead to absences or resignation from work. Indeed, many stressful situations occur in daily life. Moreover, extreme stress can cause physical and/or psychiatric disorders such as adjustment disorder and major depressive disorder (MDD), resulting in increased negative social interactions for such patients [1]. Therefore, stress management is important for a healthy life.

The immune system may contribute to the fundamental mechanisms of psychiatric disorders caused by stressful conditions, especially MDD. Under normal conditions, the hypothalamic-pituitary-adrenal (HPA) axis monitors and modulates immune balance through various hormones such as glucocorticoids [2–5]. However, stressful events induce HPA axis hyperactivity, and continuous stress leads to corticoid resistance, resulting in an excessive inflammatory response, which is a crucial risk factor for developing MDD [2, 6, 7]. A previous meta-analysis showed that serum tumor necrosis factor (TNF) α, interleukin (IL) 6, and IL18 were increased in MDD patients [8, 9]. Furthermore, TNFα and IL18 levels were greater in the hippocampus of an animal model of MDD than those in controls [10]. Therefore, stress and stress-induced psychiatric symptoms may be closely associated with the immune system, even though these direct interactions have not been clarified.

IL18 is a proinflammatory cytokine that was found to induce interferon-γ in 1995 [11]. IL18 has two forms, an active 18-kDa mature form, and a non-active 24-kDa precursor form. Cleaved caspase-1 metabolizes the precursor form of IL18 to generate the active form [12–15]. In our previous studies, we found that IL18 was associated with energy metabolism and psychiatric disorders such as depression [16–18]. Regarding neural function, IL18 exists in neural cells of the brain in a precursor form, and its deficiency leads to hippocampal impairment and a depressive-like phenotype [18, 19]. Thus, IL18 is closely related to neural function and psychiatric phenotypes such as depression as well as the immune system. However, we previously only evaluated hippocampal function under IL18 deficiency, and no treatment was examined.

Therefore, we examined the potential role of IL18 in the hippocampus in response to a single acute stress. For this purpose, we performed the following five assessments in the current study: (1) assessment of behavioral changes in IL18-deficient mice after acute stress, (2) measurement of corticosterone release and its receptor expression in the hippocampus, (3) evaluation of differences in neurogenesis and neural inflammation with regard to cytokine expression and astrocyte and microglia density, (4) comparison of comprehensive molecular differences using RNA sequencing (RNA-Seq) and the results of our previous study, and (5) examination of the behavioral changes after intracerebroventricular administration of IL18.

## Materials and methods

### Animals and stress treatment

Male *Il18*^−/−^ mice were generated on a C57Bl/6 background as described previously [20]. C57Bl/6 *Il18*^+/+^ male littermates were used as controls. All mice were housed in groups of four to five in polycarbonate cages with free access to water and standard food. The colony room was monitored and automatically maintained under the following conditions: room temperature (22 ± 1 °C), humidity (50–60%), and a 12-h light/dark cycle (lights on at 08:00 am).

Mice were divided into four groups randomly: (1) *Il18*^+/+^ mice without stress, (2) *Il18*^+/+^ mice with stress, (3) *Il18*^−/−^ mice without stress, and (4) *Il18*^−/−^ mice with stress. The stress consisted of a single restraint stress starting at 10:00 am and lasting for 6 h. During the stress, the mice could not drink water or eat food. Mice in the non-stressed groups had free access to water and food. After the stress, mice were immediately released and free for 18 h. After 18 h, mice underwent behavioral assessments or were sacrificed (Supplementary Fig. 1A).

Twelve mice per group were used for behavioral assessments, six were used for RNA-Seq, reverse transcription quantitative polymerase chain reaction (RT-qPCR), and western blotting assays, and five were used for immunostaining. An enzyme-linked immunosorbent assay (ELISA) was performed using serum samples from mice (*n* = 12 from each group). Twelve mice per group were used for the rescue experiment involving intracerebral administration of IL18. All animal experiments were conducted according to the Guide for the Care and Use of Laboratory Animals published by the National Institutes of Health, and all protocols were approved by the Animal Care Committee of Hyogo Medical University (Hyogo, Japan; approval nos. #28041, #19-030).

### Behavioral analysis

Behavioral differences between groups were assessed with an open field test (OFT), a forced swim test (FST), and a tail suspension test (TST). These assessments were performed according to previously described procedures with minor modifications [18, 21, 22].

### Open field test

Spontaneous locomotor activity in a novel environment was tested in the OFT. A cubic open field box made of transparent acrylic plates without a ceiling (45 × 45 × 45 cm) was placed in a ventilated soundproof chamber. An overhead incandescent light bulb provided room lighting of ~140–150 lx at the center of the test arena. Each mouse was placed at the center of the test chamber and could freely explore the open field arena for 10 min. Experimental sessions were conducted between 10:00 and 12:00 am every day to minimize the effects of temporal factors such as circadian rhythm. Behavior was recorded during each session and analyzed later. The distance traveled in the open field was calculated using a computerized video-based tracking system (Be Chase ver.2021, ISONIX Co. Ltd., Kobe, Japan). The total distance that mice moved in 2 min was measured.

### Forced swim test

The FST was performed to assess depressive-like behavioral changes. A cylindrical tank (height: 35 cm diameter: 18 cm) was used for the FST. The water level was 25 cm from the bottom, and the temperature was 24 to 26 °C. Mice were individually placed in the cylinder, allowed to swim freely inside for 6 min, and recorded. The software program (Be Chase ver.2021, ISONIX Co. Ltd., Kobe, Japan) calculated the duration of immobility by summing the time segments (in seconds) that the mouse swam below the specified threshold velocity of 2.8 cm/s. The optimum threshold was determined by comparing scores rated manually from video images with scores from the automated device in preliminary studies.

### Tail suspension test

The total immobility time induced by tail suspension was measured. The mice were suspended 30 cm above the floor by an adhesive tape that was placed ~1 cm from the end of the tail. This test was performed for 5 min, and movements were recorded. The video was analyzed by two observers blinded to the group allocation.

### Sample collection

Mice were euthanized under deep anesthesia induced by inhalation of 5% isoflurane (overdose). After shallow or the absence of breathing was confirmed, blood was immediately collected from the heart. Then, the hippocampi were removed, immediately placed in liquid nitrogen, and stored at –80 °C until use.

### RNA purification

Total RNA purification was performed as previously described [19]. Briefly, we extracted total RNA using an ISOGEN kit (cat. no. 311-02501, Wako Pure Chemical Industries, Ltd., Osaka, Japan), following the manufacturer’s protocol, and then treated the RNA with 5 U of RNase inhibitor and 5 U of DNase I at 37 °C for 30 min. After purification by phenol/chloroform and ethanol precipitation, total RNA was dissolved in sterilized and de-ionized distilled water.

### RNA-Seq

We outsourced the RNA-Sequencing analysis to Takara Bio Inc. (Mie, Japan). A SMART-Seq® v4 Ultra® Low Input RNA Kit for Sequencing (Illumina K.K., Tokyo, Japan) was used according to the manufacturer’s protocol. We compared the gene expression profiles of the hippocampus between stressed *l18*^+/+^ mice and stressed *Il18*^−/−^ mice (*n* = 3 per group). Details of RNA-Seq can be found on the Gene Expression Omnibus website (GSE207669). FASTQ files were aligned to the GRCm38 assembly using the DRAGEN Bio-IT Platform (Illumina). Genes with a false discovery rate < 0.3 were identified as differentially expressed genes using edgeR (Ver. 3.30.3, http://bioconductor.org/biocLite.R). A heat map was generated using the Heatplus package (Version 3.2.0, https://github.com/alexploner/Heatplus) available in Bioconductor using the regHeatmap function in the R statistical environment.

### Ingenuity pathway analysis (IPA)

IPA (QIAGEN Digital Insights, Redwood City, CA, USA) was used to analyze the functions of genes extracted by RNA-Seq as previously described [23]. Core analysis was performed to analyze the functions of extracted molecules as follows: tissue, hippocampus; all other settings, default. The network explorer of IPA was used with default settings to identify molecule-molecule interactions and pathways between molecules [19].

### RT-qPCR

RT-qPCR was performed as previously described with minor modifications [23, 24]. Briefly, total RNA (5 ng/reaction) was analyzed with the RNA-direct SYBR Green Real-Time PCR Master Mix: One-step qPCR kit (Toyobo Co. Ltd., Tokyo, Japan) following the manufacturer’s protocol. Samples were examined in duplicate reactions in 384-well plates. The median threshold cycle values were used to calculate fold changes between the groups. The following cycling protocols were used with the QuantiStudio™ 12 K Flex system (Thermo Fisher Scientific, Inc.): 30 s at 90 °C and 20 min at 61 °C for reverse transcription, followed by 45 cycles of 98 °C for 1 s, 67 °C for 15 s, and 74 °C for 35 s. Fold-change values were normalized to the glyceraldehyde-3-phosphate dehydrogenase (*Gapdh*) level using the relative standard curve method. Primer sequences for RT-qPCR are shown in Supplementary Table 1.

### Western blotting

Protein extraction from the mouse hippocampus and western blotting were performed as previously described [17, 25]. Briefly, samples were incubated in Laemmli sample buffer for 5 min at 95 °C, electrophoresed on a 10–20% sodium dodecyl sulfate polyacrylamide gel, and transferred onto polyvinylidene difluoride membranes (Hybond-P, Amersham Bioscience, Little Chalfont, UK). The primary antibodies were as follows: monoclonal rabbit anti-β-actin (cat. no. #4967; Cell Signaling Technology, Inc. (CST), Danvers, MA, USA) or anti-GAPDH (cat. no. #3683, CST) as a loading control, rabbit anti-heat shock transcription factor 5 (HSF5) polyclonal antibody (cat. No. MBS2520556, MyBioSource, San Diego, CA, USA), rabbit anti-gamma-aminobutyric acid type A receptor subunit rho1 (GABRR1) polyclonal antibody (cat. no. 25899-1-AP; Proteintech Japan, Tokyo, Japan), and rabbit anti-transthyretin polyclonal antibody (TTR; cat. no. sc-13098; Santa Cruz Biotechnology, Inc., Dallas, TX, USA). All membranes were blocked using 5% skim milk in phosphate-buffered saline containing 0.1% Triton X-100 and incubated with primary antibodies at 4 °C overnight. Then, the membranes were incubated with horseradish peroxidase-conjugated secondary antibodies (#NA9340V and #RPN1025; GE Healthcare, Amersham, UK). Antibody reactions were visualized with an LAS-4010 photo-image analyzer (Fuji Photo Film Co, Ltd, Tokyo, Japan). The density of specific protein bands was analyzed using ImageJ software (http://rsbweb.nih.gov/ij/, version 1.6), and the results were normalized to the loading control levels.

### ELISA

Serum levels of corticosterone, IL18, and TTR were measured using an ELISA. ELISA kits for corticosterone, IL18, and TTR were purchased from Enzo Life Sciences, Inc. (cat. no. ADI-900-097; Farmingdale, NY, USA), Medical & Biological Laboratories Co., Ltd. (cat no. 7625; Nagoya, Aichi, Japan), and Immunology Consultants Laboratory, Inc. (cat. no. E-90PRE; Portland, OR, USA), respectively. All ELISAs were performed according to the manufacturers’ protocols.

### Immunofluorescence staining

Immunofluorescence staining was performed to reveal differences in neurogenesis and neuroinflammation. Mice were deeply anesthetized with 5% isoflurane and transcardially perfused with 50 ml of saline and 4% periodate-lysine-paraformaldehyde fixative. Then, the fixed brains were removed. Coronal brain slices with 20 μm thickness that included the dentate gyrus of the hippocampus were prepared. These sections were incubated at 90 °C for 40 min with an antigen retrieval reagent (Histo VT One, cat. no. 06380, NACALAI TESQUE, INC. Kyoto, Japan) and blocked with 5% bovine serum albumin for 1 hour. The sections were stained overnight at 4 °C with the following antibodies: Ki67 (1:200, #550609, BD Biosciences, NJ, USA), doublecortin (DCX; 1:2000, #AB2253, EMD Millipore, Burlington, MA, USA), GFAP (1:200, cat. No. #12389, CST), and IBA1 (1:500, cat. no. #019-19741, FUJIFILM Wako Pure Chemical Corporation, Osaka, Japan). For immunofluorescence staining, fluorochrome-conjugated goat anti-guinea pig IgG H&L secondary antibody (dilution 1:200, Alexa Fluor® 488, cat. no. ab150185; Abcam, Cambridge, UK) or goat anti-rabbit IgG H&L secondary antibody (dilution 1:1000, Alexa Fluor® 594, cat. no. ab150078; Abcam) was added for 2 h at room temperature. Then, the sections were cover-slipped with Vectashield mounting medium with 4,6-diamidino-2-phenylindole (DAPI) (cat. no. H-1800; Vector Laboratories, Inc., Burlingame, CA, USA) and visualized with a confocal microscope (LSM780, Carl Zeiss Co. Ltd., Tokyo, Japan). The images were scanned using ZEN Imaging Software (Carl Zeiss Co. Ltd.) for analysis. Positive cells and their density were counted and averaged as previously described (8–10 sections/mouse) using ImageJ software [18, 26, 27]. The branch length of microglia was quantified as previously shown [28].

### Intracerebral IL18 administration

To evaluate behavioral changes in stressed *Il18*^−/−^ mice, IL18 was administered into the lateral ventricles. Mice were deeply anesthetized with isoflurane (5% for induction, 2% for maintenance), and 2 μl (1 μg/μl) of IL18 was slowly injected. The point of injection into the lateral ventricle was 2 mm lateral and 0.8 mm caudal from bregma as previously described [18]. After injection, mice were placed in their home cage for 24 h. Then, mice were subjected to stress as described in the ‘Animals and stress treatment’ section (Supplementary Fig. 1B).

### Statistical analysis

All results are expressed as means±standard error of the mean and were analyzed using Sigmaplot™ software (version 11.0; Systat Software Inc., San Jose, CA, USA). For statistical analysis of the behavioral tests, a repeated measures ANOVA with genotype (+/+ or −/−) or with stress as the between-subject factor and repeated measures (e.g., time) as the within-subject factor, was conducted on the data for each test. ELISA, RT-qPCR, western blot, and immunochemistry data were analyzed by ANOVA first, and if significance was found, the Holm-Sidak or Tukey method was applied. Differences were considered statistically significant when *p* < 0.05. All statistical analyses were performed as previously described [18].

## Results

### Behavioral changes with acute restraint stress

**Fig. 1:**
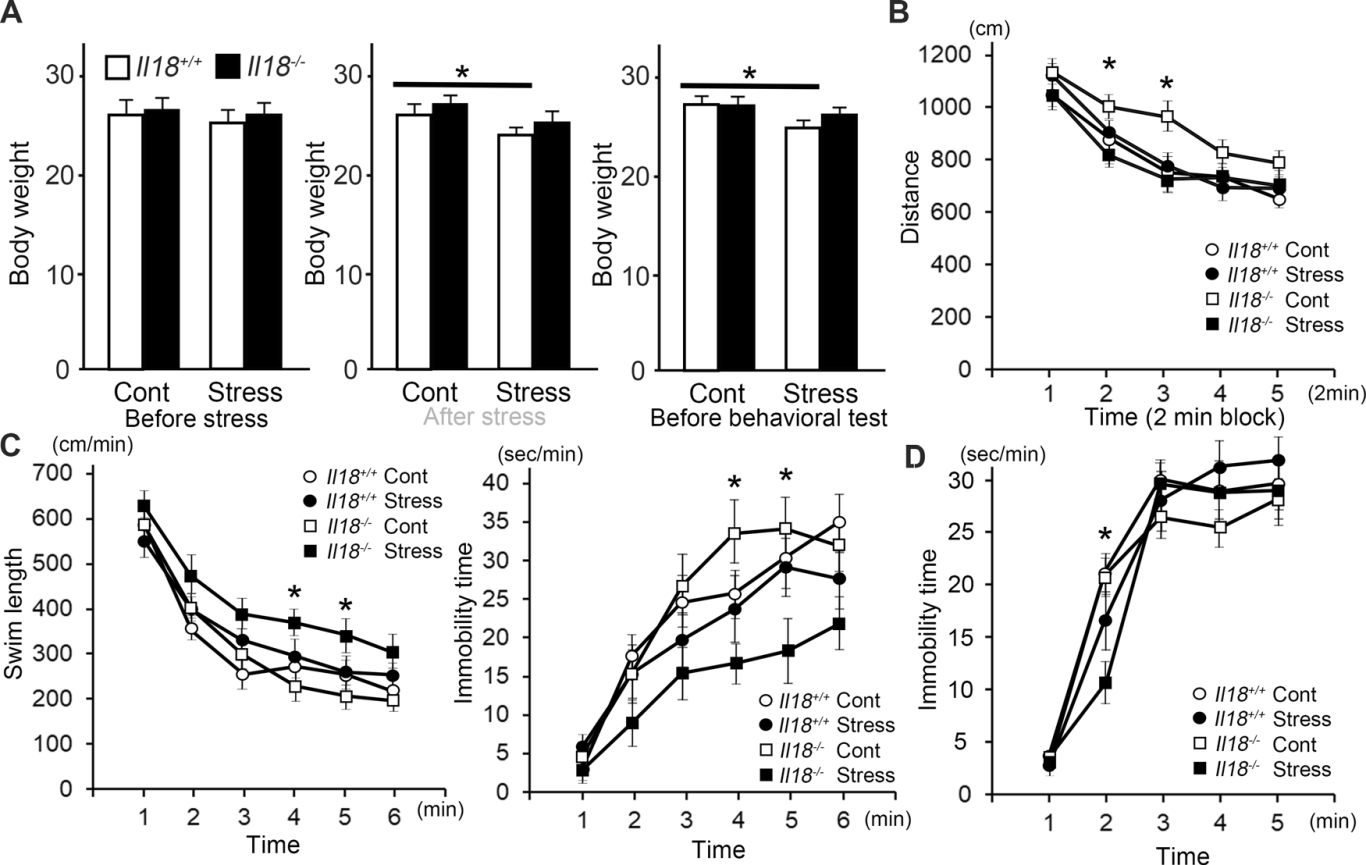
*Il18*^−/−^ mice showed behavioral changes after stress. **A** The body weights of each group before stress treatment, after treatment, and before behavioral tests are shown. The behavioral tests results, including the open field test (**B**), forced swim test (**C**), and tail suspension test (**D**), are shown. **B** The graph shows the distance traveled by each mouse during 2 min in the open field test. **C** In the forced swim test, stressed *Il18*^−/−^ mice had a longer swimming distance (left) and less immobility time (left) than those of non-stressed mice, and these phenotypes were not observed in *Il18*^+/+^ mice. **D** In the tail suspension test, stressed *Il18*^−/−^ mice showed a decreased immobility time, similar to the forced swim test. *p < 0.05 (stressed *Il18*^−/−^ mice versus non-stressed mice), (*n* = 12 per group).

The body weight of *Il18*^+/+^ mice was significantly decreased in stressed mice and remained decreased after one night, even though there was no difference between stressed and non-stressed *Il18*^−/−^ mice before stress was applied (Fig. 1A). In the OFT, the total distance traveled by *Il18*^−/−^ mice was decreased after stress; however, the distance traveled by *Il18*^+/+^ mice was not altered by stress, and no significant difference was observed between stressed *Il18*^−/−^ mice and *Il18*^+/+^ mice (Fig. 1B). In the FST, *Il18*^−/−^ mice exhibited a significant increase in swimming distance and a decrease in immobility time after stress, and these effects were not observed in *Il18*^+/+^ mice (Fig. 1C). In the TST, mice exhibited behavioral changes similar to those in the FST; the immobility time was only slightly decreased at 2 min but this was still statistically significant (Fig. 1D).

### Inflammatory response in the hippocampus after stress

**Fig. 2:**
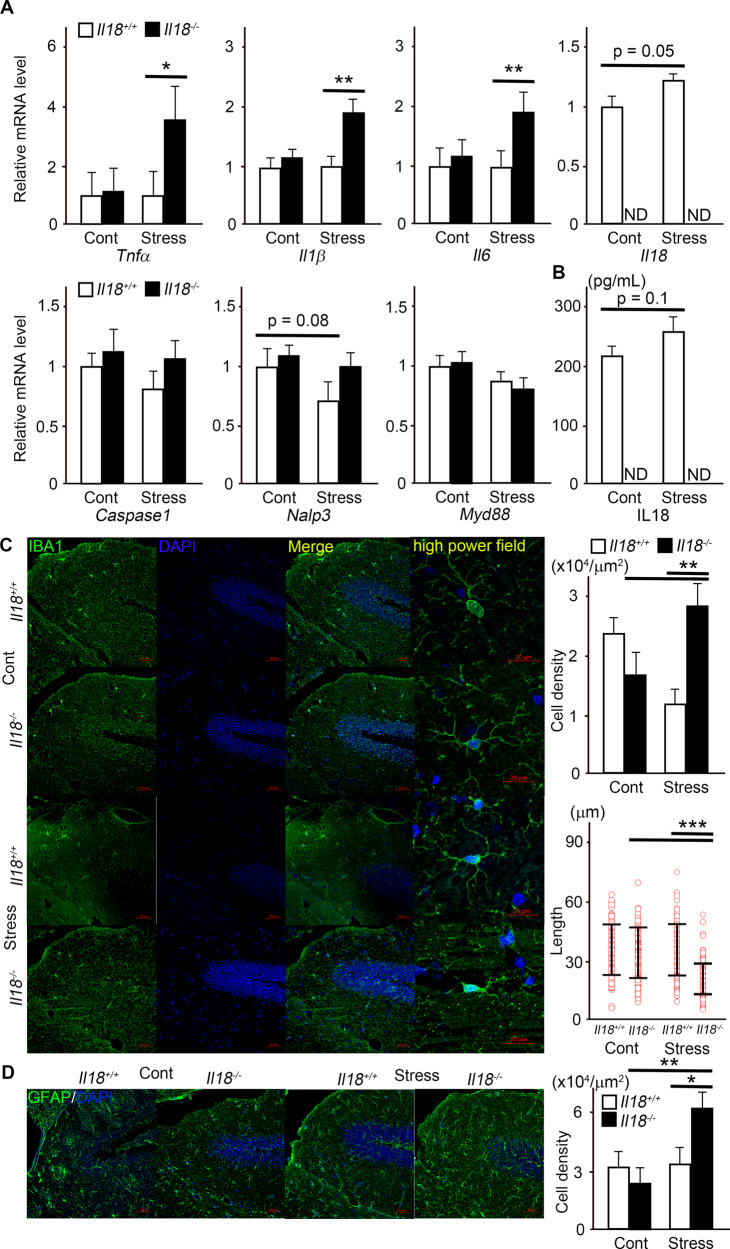
Neural inflammation in the hippocampus remained longer in *Il18*^−/−^ mice exposed to a single stress than that in *Il18*^+/+^ mice. **A** The relative mRNA expression levels of inflammatory-related genes are shown. **B** The serum IL18 level was measured in each group. The immunofluorescence staining results for IBA1 (**C**) and GFAP (**D**) are shown. The cell density and branch length of microglia (**C**) and astrocytes (**D**) are shown on the right side. *p < 0.05, **p < 0.01, ***p < 0.001. (**A**; *n* = 6 per group, **B**; *n* = 12 per group, **C**; cell density; length; *n* = 4 per group, **D**; cell density; *n* = 4 per group).

Inflammatory cytokine expression was measured to evaluate the inflammatory response after acute restraint stress. The mRNA expression levels of *Tnfα*, *IL1β*, and *Il6* were significantly increased in *Il18*^−/−^ mice after stress but were not increased in *Il18*^+/+^ mice (Fig. 2A). However, Il10 expression was not significantly different and *Il18* expression was slightly increased in *Il18*^+/+^ mice after stress (Fig. 2A, data not shown). Contrary to our predictions, no significant difference in inflammasome-related molecules, including *Caspase1*, *Nalp3*, and *Myd88*, was observed between stressed *Il18*^−/−^ mice and *Il18*^+/+^ mice (Fig. 2A). Although the same tendency as that of IL18 expression in serum was observed, i.e., a slight but not significant increase in *Il18*^+/+^ mice after stress (Fig. 2B), the serum IL1β and IL6 levels were under the detection limit (data not shown). We next examined neural inflammation in the hippocampus based on increases in inflammatory cytokines in *Il18*^−/−^ mice after stress. IBA1-positive cells were significantly increased in *Il18*^−/−^ mice after stress, and shortened microglia branches could be observed only in *Il18*^−/−^ mice after stress (Fig. 2C). Moreover, similar to IBA1, the number of GFAP-positive cells was also increased only in *Il18*^−/−^ mice after stress (Fig. 2D).

### Glucocorticoid receptors and neurogenesis in the hippocampus

**Fig. 3:**
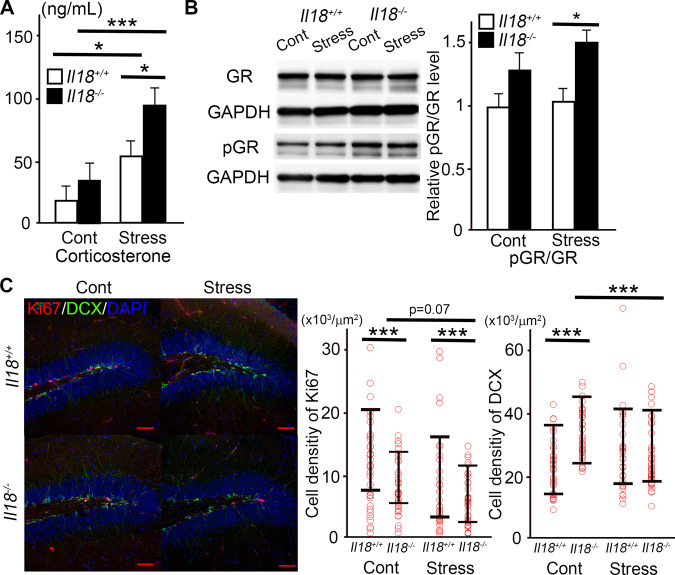
The assessment of glucocorticoid signaling and neurogenesis mediated by a single stress. **A** The serum corticosterone level in each group is shown. **B** The ratio of glucocorticoid receptors and phosphorylated glucocorticoid receptors in stressed *Il18*^−/−^ mice was increased compared with that in other groups. **C** Immunofluorescence staining of Ki67 and doublecortin (DCX) is shown, and the graphs indicate the density of Ki67- and DCX-positive cells in each group. (red; Ki67, DCX; green, blue; DAPI) *p < 0.05, ***p < 0.001. (**A**; *n* = 12, **B**; *n* = 6, **C**; *n* = 4 per group).

To evaluate the stress response, the stress-response hormone corticosterone was measured in both the control and stressed groups. As described above, serum was extracted 18 hours after restraint stress was completed. Corticosterone was significantly increased after stress in both *Il18*^+/+^ and *Il18*^−/−^ mice, and the corticosterone level in stressed *Il18*^−/−^ mice was higher than that in stressed *Il18*^+/+^ mice (Fig. 3A). The expression of phosphorylated and non-phosphorylated glucocorticoid receptor was compared as shown in Fig. 3B, and a significant increase in the phosphorylated glucocorticoid receptor/glucocorticoid receptor ratio was observed in stressed *Il18*^−/−^ mice but not in stressed *Il18*^+/+^ mice. Upregulated glucocorticoid signals can induce inhibition of hippocampal neurogenesis [29–31]. Therefore, we next assessed hippocampal neurogenesis using immunohistochemistry according to our previous study [18]. Significantly fewer Ki67-positive cells were observed in*Il18*^−/−^ mice than in *Il18*^+/+^ mice regardless of whether stress was induced (Fig. 3C). Moreover, DCX-positive cells were significantly increased in non-stressed *Il18*^−/−^ mice compared with those in *Il18*^+/+^ mice, and the number of cells decreased after stress. However, no difference was observed between stressed *Il18*^+/+^ and *Il18*^−/−^ mice (Fig. 3C).

### RNA-Seq and IPA

**Fig. 4:**
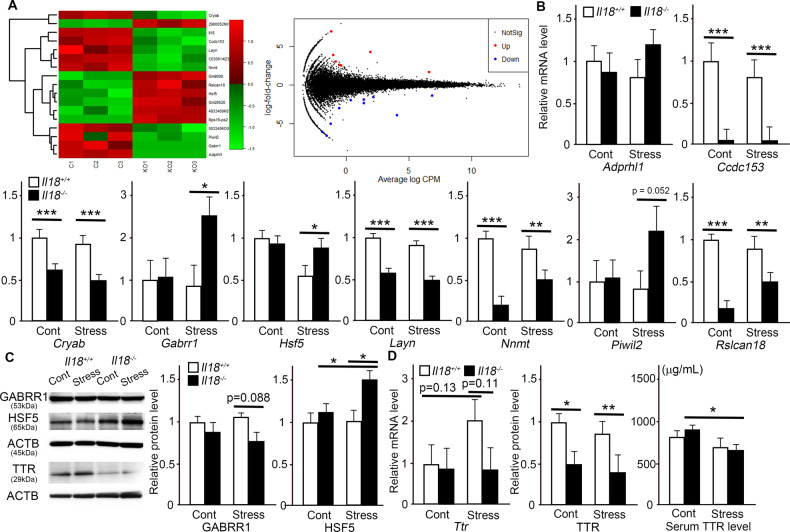
Identification of specific molecules in *Il18*^−/−^ mice that respond to acute stress. **A** The heatmap (left) and volcano map (right) generated from RNA sequencing are shown. (**C**; stressed *Il18*^+/+^ mice, KO; stressed *Il18*^−/−^ mice). **B** The reverse transcription quantitative polymerase chain reaction results are shown. The molecules were extracted from RNA sequencing as shown in Table 1. **C** The protein expression of gamma-aminobutyric acid type A receptor subunit rho1 (GABRR1), heat shock transcription factor 5 (HSF5), and transthyretin (TTR) is shown. **D** The mRNA, protein, and serum levels of TTR/Ttr are shown. *p < 0.05, **p < 0.01, ***p < 0.001. (**A**; *n* = 3, **B**–**D**; n = 6, enzyme-linked immunosorbent assay *n* = 12 per group).

RNA-Seq was performed to identify the genes that responded specifically in stressed *Il18*^−/−^ mice (GSE207669). The results are shown as a volcano map in Fig. 4A. Seventeen genes with a false discovery rate <0.30 were extracted from the RNA-Seq results based on previous study [32, 33], and nine genes were assigned GenBank accession numbers (Table 1, Supplementary Table 2). RT-qPCR was performed to confirm the RNA-Seq results (Fig. 4B). According to the RT-qPCR results, *Gabrr1* and *Hsf5* specifically responded in stressed *Il18*^−/−^ mice (Fig. 4B). We next verified their protein expression by western blotting. The GABRR1 expression level in stressed *Il18*^−/−^ mice was slightly decreased compared with that in *Il18*^+/+^ mice, but the result was not significant and was opposite to the RT-qPCR result (Fig. 4B, C). However, HSF5 expression in stressed *Il18*^−/−^ mice was significantly upregulated compared with that in the other groups, and this was only observed in stressed *Il18*^−/−^ mice (Fig. 4C).

**Table 1:** Nine genes with a false discovery rate <0.30 were extracted from the RNA sequencing results of *Il18*-deficient mice and assigned GenBank accession numbers.

Gene symbol	Entrez gene name	GenBank ID
*Adprhl1*	ADP-ribosylhydrolase like 1	NM_172750
*Ccdc153*	coiled-coil domain containing 153	NM_001081369
*Cryab*	crystallin, alpha B	NM_009964
*Gabrr1*	gamma-aminobutyric acid (GABA) C receptor, subunit rho 1	NM_008075
*Hsf5*	heat shock transcription factor family member 5	NM_001045527
*Il18*	interleukin 18	NM_008360
*Layn*	layilin	NM_001033534
*Nnmt*	nicotinamide N-methyltransferase	NM_010924
*Piwil2*	piwi-like RNA-mediated gene silencing 2	NM_021308
*Rslcan18*	regulator of sex-limitation candidate 18	NM_001256052

Moreover, we searched for interacting pathways between molecules extracted from the RNA-Seq results and immune-related genes in IL1β and IL6 pathways as shown in Fig. 2A. As shown in Supplementary Fig. 2, genes highlighted in yellow are related to IL18, and genes highlighted in blue are related to molecules extracted from RNA-Seq. To explore the functions and annotations of extracted molecules, IPA core analysis was performed as shown in Supplementary Table 3.

Based on our previous study, we also examined TTR expression. The RNA-Seq results showed that TTR expression in stressed *Il18*^−/−^ mice was decreased compared with that in stressed *Il18*^+/+^ mice (*p* < 0.001). Although mRNA expression was not significantly altered, a significant decrease in TTR was observed at the protein level (Fig. 4C, D). Moreover, although these results were not specifically shown in stressed *Il18*^−/−^ mice, a significant decrease in the serum TTR level was specifically observed in stressed *Il18*^−/−^ mice (Fig. 4D).

### Recovery of behavioral changes induced by intracerebral IL18administration

**Fig. 5:**
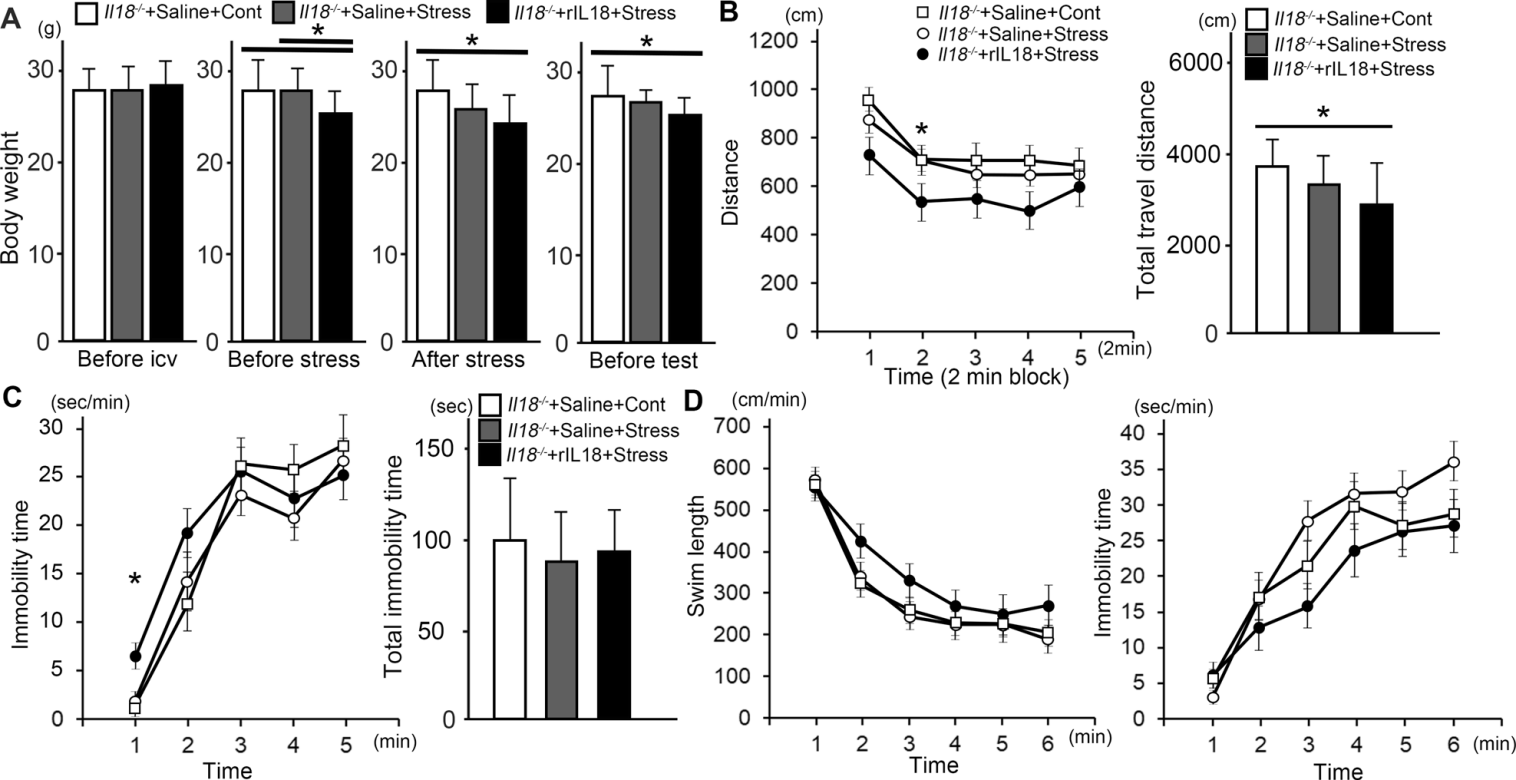
Intracerebral injection of IL18 partially improved behavioral changes in stressed *Il18*^−/−^ mice. **A** The body weights of each group before intracerebral injection of IL18, before stress treatment, after treatment, and before behavioral tests are shown. The behavioral tests results, including the open field test (**B**), tail suspension test (**C**), and forced swim test (**D**), are shown. **B** The graph shows the distance traveled by each mouse during 2 min (left) and the total distance (right) traveled in the open field test. **C** In the tail suspension test, a significant difference was found only during the first min. **D** In the forced swim test, no significant difference was observed. *p < 0.05 (stressed *Il18*^−/−^ mice with IL18 administration versus the other groups), (*n* = 12 per group).

Intracerebral IL18 administration was performed to determine whether behavioral changes in stressed *Il18*^−/−^ mice could be reversed. Intracerebral injection of IL18 induced significant decreases in body weight in stressed *Il18*^−/−^ mice (Fig. 5A). In the OFT, stressed *Il18*^−/−^ mice injected with IL18 exhibited significantly less distance traveled than that in other groups (Fig. 5B). Contrary to our predictions, only a slight influence on recovery of stressed *Il18*^−/−^ mice injected with IL18 was observed in the TST (Fig. 5C) and FST (Fig. 5D).

## Discussion

In this study, we investigated the function of IL18 in neural cells and the immune system under acute stress and found the following results: (1) stressed *Il18*^−/−^ mice showed hyperactivity in behavioral assessments compared with other groups; (2) neural inflammation induced by a single stress remained much longer in IL18-deficient mice, suggesting that IL18 may modulate the stress response; (3) stressed *Il18*^−/−^ mice exhibited higher serum corticosterone and glucocorticoid receptor phosphorylation levels in the hippocampus resulting in a slight suppression of neurogenesis; (4) specific genes such as *HSF5* and *TTR* were extracted from stressed *Il18*^−/−^ mice in the molecular analysis; and (5) the behavioral changes induced by acute stress were slightly attenuated by intracerebral IL18 administration.

The FST and TST are common methods used to assess depression-like phenotypes [34, 35]. Typically, an increased immobility time and decreased swim distance indicate depressive symptoms in animals [34, 35]. However, some reports have suggested that the opposite behavioral changes, i.e., decreased immobility and increased swimming distance, are observed when mice exhibit anxiety and fear [36, 37]. Moreover, Yamada et al (2000) reported that mice showed less immobility in the FST because of ‘inappropriate coping responses’ [37]. In our behavioral results shown in Fig. 1, the immobility time of stressed *Il18*^−/−^ mice in the FST and TST was significantly increased, which was not observed in stressed *Il18*^+/+^ mice. Therefore, our results suggested that *Il18*^−/−^ mice might exhibit behavioral changes including heightened anxiety in response to acute restraint stress because of decreased stress tolerance, and they may be regarded as a diathesis-stress model.

Inflammatory cytokines and neural inflammation are major factors that mediate depression and anxiety. To examine the relationship between the behavioral changes in Fig. 1 and the inflammatory response linked to IL18, we measured inflammatory and anti-inflammatory cytokine expression. As shown in Fig. 2A, *Tnfα*, *Il1β*, and *Il6* expression in stressed *Il18*^−/−^ mice was significantly greater than that in the other groups. Moreover, neuroinflammation in the hippocampus was assessed by staining for IBA1, a microglial marker, and GFAP, an astrocyte marker. According to Fig. 2C, D, the numbers of GFAP- and IBA1-positive cells were significantly increased in stressed *Il18*^−/−^ mice compared with those in the other groups. In addition, the active microglia in stressed *Il18*^−/−^ mice had a reduced total process length. In several models of depression, the expressions of inflammatory cytokines such as *Tnfα*, *Il1b*, and *Il6* in the hippocampus were increased and higher microglial activation in the hippocampus was observed compared with controls [38–41]. In summary, neural inflammation continued for a prolonged time in *Il18*^−/−^ mice but was not observed in *Il18*^+/+^ mice at 18 h after stress, suggesting that IL18 plays a role in stress-mediated inflammatory regulation.

Stress induces HPA axis hyperactivity [6], and corticosterone is a major hormone that immediately responds to stress [42]. Chronic glucocorticoid exposure induces inhibition of neurogenesis in the dentate gyrus, resulting in depression and anxiety disorder [43–45]. However, acute stress, such as 3 h of immobilization stress, can induce an increase in corticosterone, resulting in increased neurogenesis in the dentate gyrus of rats [46]. During neurogenesis in the dentate gyrus, stress-induced corticosterone activates microglia to maintain homeostasis of neurogenesis in the hippocampus [47]. Moreover, microglia can induce factors to modulate neural proliferation and survival [48]. Glucocorticoid-induced activation of glucocorticoid receptors in the hippocampus mediates microglial activation, resulting in hippocampal neuroinflammation and depressive-like behaviors [49].

As shown in Fig. 3A, the serum corticosterone level was not different between non-stressed *Il18*^+/+^ and *Il18*^−/−^ mice. However, serum corticosterone was significantly increased in both stressed *Il18*^+/+^ and *Il18*^−/−^ mice compared with that in non-stressed control mice. In addition, the corticosterone level was much higher in stressed *Il18*^−/−^ mice than that in stressed *Il18*^+/+^ mice. In stressed *Il18*^−/−^ mice, glucocorticoid receptor phosphorylation was significantly increased compared with that in non-stressed mice (Fig. 2B), which is a result of the increased corticosterone level [50]. As shown in Fig. 3C, although the number of DCX-positive cells was not different between the stressed groups, the number of Ki-67-positive cells was significantly decreased in *Il18*^−/−^ mice. In addition, the number of Ki-67-positive cells in stressed *Il18*^−/−^ mice was slightly but not significantly decreased compared with that in non-stressed mice. Neurogenesis was assessed 1 day after stress application, and more time may be required to determine the effects on neurogenesis because granule cells in the dentate gyrus require more than 2 weeks to mature [51]. In summary, our results suggested that the longer duration of neural inflammation in the hippocampus of stressed *Il18*^−/−^ mice was related to increased corticosterone levels and might exert a significant influence on neurogenesis. Moreover, our results also suggested that these mice require a longer time to attenuate stress-induced neuroinflammation and return to a homeostatic state.

RNA-Seq was performed to identify molecules specifically expressed in IL18-deficient mice exposed to stress. Figure 4A and Table 1 show nine molecules that were identified. RT-qPCR was performed to determine whether these molecules were specifically associated with stress in IL18-deficient mice. Based on this analysis, *Gabrr1* and *Hsf5* were suggested to specifically respond to stress in *Il18*^−/−^ mice (Fig. 4B). Then, their protein expression was confirmed by western blotting (Fig. 4C), and HSF5 was shown to be significantly upregulated, similar to the RT-qPCR results. HSFs are indispensable factors that respond to acute stress [52]. However, to our knowledge, no reports have described the relationship among HSF5, stress, and psychiatric disorders. Thus, these results were novel and suggested that HSF5 might be a critical factor that regulates the stress response along with IL18.

However, TTR/Ttr was decreased in *Il18*^−/−^ mice compared with that in *Il18*^+/+^ mice. According to our previous study, differences in *Ttr* are commonly detected in the liver, brown adipose tissue, hippocampus, and prefrontal cortex [16–19]. Although mRNA expression of Ttr was not significantly altered, its protein expression in the hippocampus was significantly decreased in both stressed *Il18*^+/+^ and *Il18*^−/−^ mice compared with that in non-stressed mice. In a clinical study, TTR was decreased in the cerebrospinal fluid of depressed patients compared with that in healthy controls [53]. Moreover, previous reports have suggested that TTR is a major factor in resistance to stress exposure [53, 54]. Although the serum TTR level was not different in *Il18*^+/+^ and *Il18*^−/−^ mice, the TTR level in the hippocampus of *Il18*^−/−^ mice was decreased, and this was not affected by acute stress. Therefore, these results suggested that TTR might play an important role in mediating the stress response in IL18-deficient mice.

Intracerebral administration of IL18 was performed to determine whether behavioral changes in stressed *Il18*^−/−^ mice could be reversed. Contrary to our prediction, no difference was observed between stressed *Il18*^−/−^ mice administered saline or IL18 (Fig. 5). However, the increased travel distance observed in stressed *Il18*^−/−^ mice was decreased by IL18 administration (Fig. 5B). No significant effect was observed in the TST and FST, although a slight recovery of behavioral effects was observed (Fig. 5C, D). Intracerebral injection, including saline, increased corticosterone levels in mice, and intracerebral injection itself acted as a source of stress in mice [55]. Therefore, our results suggested that intracerebral administration might be equivalent to a single restraint stress treatment and could produce the behavioral results shown in Fig. 5B, C. However, IL18 injection had a partial protective effect against stress as shown in Fig. 5A.

In summary, we found that stressed *Il18*^*−/−*^ mice exhibited hyperactivity in behavioral assessments, prolonged stress-induced neural inflammation, increased levels of serum corticosterone and glucocorticoid receptor phosphorylation in the hippocampus, expressions of specific genes including *HSF5* and *TTR*, and the amelioration of acute stress-induced behavioral changes after the intracerebral administration of IL18. These results suggest that IL18 may modulate the stress response and maintain a balance between neural inflammation and glucocorticoid signaling.

Regarding the limitations of our study, only a single stress was performed. Continuous stress treatment is warranted in future studies to develop other models of psychiatric disorders and major depressive disorders. Moreover, all analyses were performed one night after the stress was administered. A study of the time course of behavioral and neuro inflammatory changes is warranted. IL18 has pleiotropic functions. IL18 was increased and peripheral IL18 was negatively correlated with brain activity in depressed patients [9, 56]. Moreover, the administration of IL18 into the amygdala, but not the hippocampus, led to severe depression-like behaviors [57]. However, a previous study indicated that *Il18*^*−/−*^ mice exhibited morphological changes resulting in impaired learning and memory, and depressive-like behavioral changes [18]. further analyses of the role of IL18 in different brain regions such as the amygdala and striatum and brain cells including neural cells and astrocytes are warranted.

## Conclusion

We investigated the role of IL18 in stress. First, we revealed that IL18 deficiency resulted in behavioral changes such as stress-induced hyperactivity. Then, we found that stress-mediated neural inflammation was maintained in *Il18*^*−/−*^ mice through the augmentation of glucocorticoid signaling, resulting in a slight attenuation of neurogenesis. In addition, *HSF5* and *TTR* might be the genes responsible for specific stress responses in IL18-deficient mice. Finally, we demonstrated a partial recovery effect produced by intracerebral IL18 administration. IL18 might be an indispensable cytokine that modulates and maintains homeostasis in the brain during stress. Moreover, our study suggests that IL18 may be a therapeutic target for psychiatric disorders, including stress-related disorders, although further clarification of its potential regulatory mechanism is required.

## Supplementary information

Supplementary Table 1

Supplementary Table 2

Supplementary Table 3

Supplementary Figure 1

Supplementary Figure 2

Supplementary figure legends

## Data Availability

The datasets shown and/or analyzed in the present study are available from the corresponding author upon reasonable request.
