# Supplementary material for: Acute stress induces severe neural inflammation and overactivation of glucocorticoid signaling in interleukin-18-deficient mice
Source: Transl Psychiatry. 2022 Sep 23;12:404. doi: 10.1038/s41398-022-02175-7 (PMC9508168; doi:10.1038/s41398-022-02175-7)
Supplement: Supplementary file 1 — Supplementary Table 1 [file 41398_2022_2175_MOESM1_ESM.docx]

Table S1. Primer sequences for all genes used in this study.

| **Symbol** | **Genbank ID** | **Primer Sequences, 5'-3'**  **(Forward/Reverse)** |
| --- | --- | --- |
| *Adprh1* | NM_172750 | AGCTGCCACCAAAGCG |
|  |  | AGCGTCTCCAGCCGTTC |
| *Caspase1* | NM_009807.2 | CTGTATTCACGCCCTGTTGGA |
|  |  | GGCGTTTCTTTTCTACACCGCAGA |
| *Ccdc153* | NM_001081369 | AAGCCTCACTGGGATGCAAA |
|  |  | AAATTGCCGCAGTTGCTCC |
| *Cryab* | NM_009964 | TAATAAAACCCCTGACCTCACCATTCCA |
|  |  | CCGGCCCCTTATATATGCAGT |
| *Gabrr1* | NM_008075 | TTCCCCAGCTCCAACAACC |
|  |  | AGATCTTCTTTACAAGTCGGCCAT |
| *Hsf5* | NM_001045527 | GGCTACCATTCAGCACACCG |
|  |  | ATTTGTTCTGAAGGCAAGATATTCTCCGA |
| *Il1b* | NM_008361.4 | CAAGGAGAACCAAGCAACGACAAAA |
|  |  | GGTGGGTGTGCCGTCTTTCATTAC |
| *Il6* | NM_009778 | ACAAGTCGGAGGCTTAATTACACA |
|  |  | CACAACTCTTTTCTCATTTCCACGAT |
| *Il18* | AY362457.1 | AAGTGCCAGTGAACCCCAGACCA |
|  |  | CACAGAGAGGGTCACAGCCAGTCC |
| *Layn* | NM_001033534 | GCCAGCAACACCCTTACTTCC |
|  |  | CAAAGCAGCCTCTCTTCGTT |
| *Myd88* | NM_010851.3 | TCCGACCGTGACGTCCT |
|  |  | ACCATGCGGCGACACC |
| *Nalp3* | AY355340.1 | CCTCTTCCTCATGGATGGCT |
|  |  | CCTCCCCAATGTGCTCGTCA |
| *Nnmt* | NM_010924 | AGCTCTAAGACTCCAAAAGCCAAC |
|  |  | TGCACCAGCCTGATGCTC |
| *Piwil2* | NM_021308 | CCGTGTTCTGACCTGTGCAT |
|  |  | CTCCCCACAAGCTTCATATCCAG |
| *Pscan18* | NM_001256052 | TACAGCAGGAGTTATCACCTCAGTCG |
|  |  | TTTGCACTTGTAGGATTTCTGTCCACT |
| *Tnfa* | AY423855.1 | CCTCTTCTCATTCCTGCTTGTGG |
|  |  | ATCACCCCGAAGTTCAGTAGACA |
