# Supplementary material for: Acute stress induces severe neural inflammation and overactivation of glucocorticoid signaling in interleukin-18-deficient mice
Source: Transl Psychiatry. 2022 Sep 23;12:404. doi: 10.1038/s41398-022-02175-7 (PMC9508168; doi:10.1038/s41398-022-02175-7)
Supplement: Supplementary file 2 — Supplementary Table 2 [file 41398_2022_2175_MOESM2_ESM.docx]

Supplementary Table 2. A total of 17 genes with a false discovery rate less than 0.30 were extracted from the RNA sequencing results of *Il18-*deficient mice.

| Gene symbol | Entrez Gene Name | GenBank ID |
| --- | --- | --- |
| *Adprhl1* | ADP-ribosylhydrolase like 1 | NM_172750 |
| *Ccdc153* | coiled-coil domain containing 153 | NM_001081369 |
| *Cryab* | crystallin, alpha B | NM_009964 |
| *C030014I23Rik* | RIKEN cDNA C030014I23 gene | none |
| *Gabrr1* | gamma-aminobutyric acid (GABA) C receptor, subunit rho 1 | NM_008075 |
| *Gm26520* | predicted gene, 26520 | none |
| *Gm9008* | predicted pseudogene 9008 | none |
| *Hsf5* | heat shock transcription factor family member 5 | NM_001045527 |
| *Il18* | interleukin 18 | NM_008360 |
| *Layn* | layilin | NM_001033534 |
| *Nnmt* | nicotinamide N-methyltransferase | NM_010924 |
| *Piwil2* | piwi-like RNA-mediated gene silencing 2 | NM_021308 |
| *Rps19-ps2* | ribosomal protein S19, pseudogene 2 | none |
| *Rslcan18* | regulator of sex-limitation candidate 18 | NM_001256052 |
| *2900052N01Rik* | RIKEN cDNA 2900052N01 gene | none |
| *4933409K07Rik* | RIKEN cDNA 4933409K07 gene | none |
| *5033406O09Rik* | RIKEN cDNA 5033406O09 gene | none |
