# Supplementary material for: Acute stress induces severe neural inflammation and overactivation of glucocorticoid signaling in interleukin-18-deficient mice
Source: Transl Psychiatry. 2022 Sep 23;12:404. doi: 10.1038/s41398-022-02175-7 (PMC9508168; doi:10.1038/s41398-022-02175-7)
Supplement: Supplementary file 3 — Supplementary Table 3 [file 41398_2022_2175_MOESM3_ESM.docx]

Table S3. Disease association or functional annotation based on Ingenuity Pathway Analysis.

| **Diseases or Functions Annotation** | **p-value** | **Molecules** |
| --- | --- | --- |
| Development of GABAergic synapse | 0.00152 | *Npas4* |
| Neuropathy of hippocampus | 0.00152 | *Il18* |
| Outgrowth of neurites | 0.0024 | *Limk1,Npas4* |
| Neuronal cell death | 0.0135 | *Npas4,Ttr* |
| Outgrowth of axons | 0.0256 | *Limk1* |
| Abnormal morphology of nervous system | 0.0256 | *Limk1,Ttr* |
| Synaptic transmission of hippocampal CA1 region | 0.033 | *Il18* |
| Loss of neurons in central nervous system | 0.0359 | *Ttr* |
| Quantity of synapse | 0.0403 | *Npas4* |
