# Supplementary figures and images for: Acute stress induces severe neural inflammation and overactivation of glucocorticoid signaling in interleukin-18-deficient mice
Source: Transl Psychiatry. 2022 Sep 23;12:404. doi: 10.1038/s41398-022-02175-7 (PMC9508168; doi:10.1038/s41398-022-02175-7)

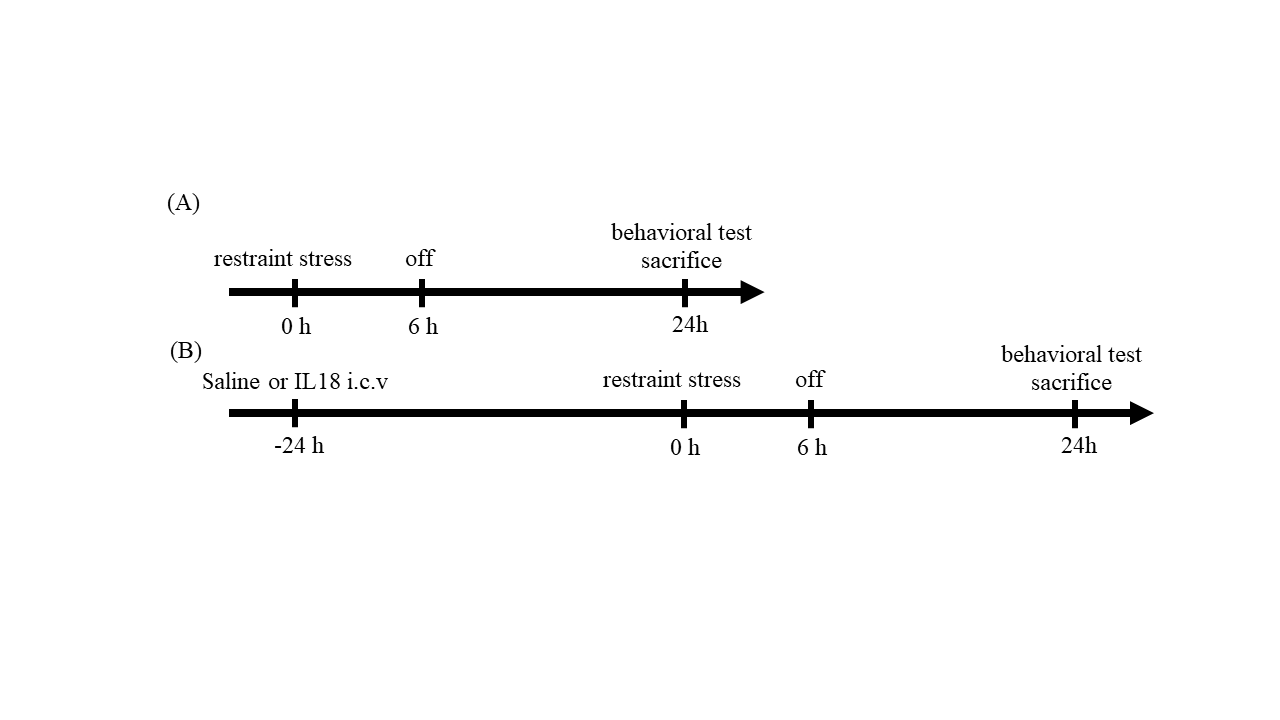

Supplement: Supplementary file 4 — Supplementary Figure 1 [file 41398_2022_2175_MOESM4_ESM.tif]

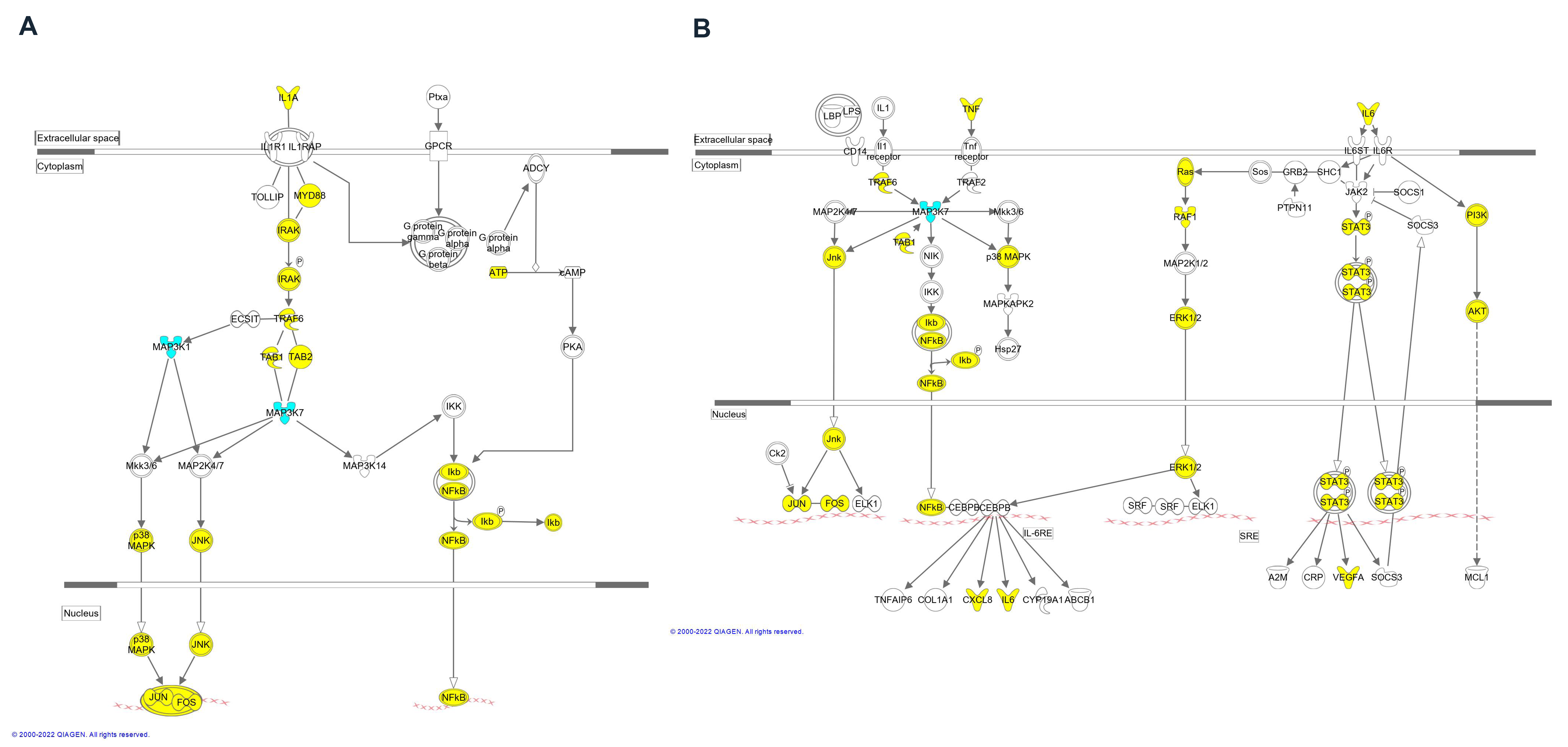

Supplement: Supplementary file 5 — Supplementary Figure 2 [file 41398_2022_2175_MOESM5_ESM.tif]
