# Supplementary material for: Acute stress induces severe neural inflammation and overactivation of glucocorticoid signaling in interleukin-18-deficient mice
Source: Transl Psychiatry. 2022 Sep 23;12:404. doi: 10.1038/s41398-022-02175-7 (PMC9508168; doi:10.1038/s41398-022-02175-7)
Supplement: Supplementary file 6 — Supplementary figure legends [file 41398_2022_2175_MOESM6_ESM.docx]

Supplementary Figure 1. Study schedule.

Supplementary Figure 2. Ingenuity pathway analysis of molecules extracted from RNA sequencing and the (A) IL1β and (B) IL6 signaling pathways.
